# Health Tract, No. 281

**Published:** 1867-03

**Authors:** 


HEALTH TRACT, No. 281.

### CONSUMPTIVE LOCALITIES

Not those which cause consumption, but which prevent or tend to cure it. Whatever represses the action of the lungs, as grief, or binding clothing, tends to develop Phthisis; whatever expands them, wards it off, on the principle that the larger the lungs are, the better is the blood purified, because more air is consumed—that is, more oxygen is conveyed to the blood, and oxygen is its life. All know that the narrow chested are more liable to the disease than those who have well developed lungs—not so much that the latter had more lungs originally, but that from habits of life the now good pair of lungs were exercised, more developed, were brought to their full capacity, thus enabling them to receive a much larger amount of air at each breath than would have been the case otherwise.

A bladder partly filled with air, will soon distend to bursting if held near the fire, because the heat rarifies the air, and makes a given amount occupy a larger space. The higher we ascend above the level of the sea, the more rarified the air becomes, and distends the lungs more fully; but this given quantity being less nutritious than an equal bulk of common air, the lungs make instinctive efforts to take in more, and this has the effect to give the lungs a permenently fuller development. If half an hour daily is expended in taking in forcible, full and deep inspirations, the circumference of the chest is perceptibly increased. Besides, the air of elevated situations being purer, is more nutritious, gives more life and vigor to the system. All know that mountain air is purer than that of the plain, and that mountaineers are more healthy than those who live on flat lands. About one person in six dies of consumption in England and the United States. In the City of Mexico, seven thousand feet above the level of the sea, about one person in every hundred dies of consumption. In the higher Alps it is almost absent. It is a rare occurrence among the Priests on the great St. Bernard, and is scarcely ever observed amongst the inhabitants of the upper Alps and when they do become consumptive away from home, and return to their native mountains before the disease has made great progress, they are generally cured. The further we go north, the less elevation is required to almost banish consumption. In the tropics, it is rare above seven thousand feet; in the temperate zone, it is rare above four thousand feet; in Switzerland, between forty-six and forty-eight degrees, North Latitude, its frequency diminishes above three thousand feet; in the Black Forest, between forty-seven and forty-nine degrees, North Latitude, above two thousand five hundred feet; in the Hazy Mountains, and those of Thüringen and Selicia, between fifty and fifty-two degrees, North Latitude, at an elevation of one thousand five hundred feet, consumption is almost unknown.

Brehmer assures us that he has never seen tubercular consumption in the Görbersdörf at one thousand seven hundred feet. Birds which live most in the higher regions have the largest lungs. The great practical inference is, whatever promotes a full and deep breathing, tends to the arrest and cure of consumption, whether it be by breathing a rarified air in elevated localities, or to promote deep breathing by artificial means; and these have been our principles of treating consumption for more than twenty-five years.

